# A functional polymorphism in the *IL1B *gene promoter, *IL1B *-31C>T, is not associated with cerebral malaria in Thailand

**DOI:** 10.1186/1475-2875-4-38

**Published:** 2005-08-14

**Authors:** Jun Ohashi, Izumi Naka, Akihiro Doi, Jintana Patarapotikul, Hathairad Hananantachai, Noppadon Tangpukdee, Sornchai Looareesuwan, Katsushi Tokunaga

**Affiliations:** 1 Department of Human Genetics, Graduate School of Medicine, The University of Tokyo,7-3-1 Hongo, Bunkyo-ku, Tokyo 113-0033, Japan; 2 Faculty of Tropical Medicine, Mahidol University, 420/6 Rajvithi Road, Bangkok, Thailand

## Abstract

**Background:**

IL-1β and IL-1RA levels are higher in the serum of cerebral malaria patients than in patients with mild malaria. Recently, the level of *IL1B *expression was reported to be influenced by a polymorphism in the promoter of *IL1*, *IL1B *-31C>T.

**Methods:**

To examine whether polymorphisms in *IL1B *and *IL1RA *influence the susceptibility to cerebral malaria, *IL1B *-31C>T, *IL1B *3953C>T, and *IL1RA *variable number of tandem repeat (VNTR) were analysed in 312 Thai patients with malaria (109 cerebral malaria and 203 mild malaria patients).

**Results:**

In this population, *IL1B *-31C>T and *IL1RA *VNTRwere detected, while *IL1B *3953C>T (i.e., *IL1B *3953T) was not observed in the polymorphism screening for 32 patients. Further analyses for *IL1B *-31C>T and *IL1RA *VNTR in 110 cerebral malaria and 206 mild malaria patients showed no significant association of these polymorphisms with cerebral malaria.

**Conclusion:**

The present results suggest that *IL1B *-31C>T and *IL1RA *VNTR polymorphisms do not play a crucial role in susceptibility or resistance to cerebral malaria.

## Introduction

Interleukin 1 (IL-1) is a proinflammatory cytokine that has been suggested to play a crucial role in the pathogenesis of cerebral malaria [1]. Histopathology and immunohistologic studies detected IL-1β in the brain and liver from a case of fatal cerebral malaria [2]. Furthermore, expression of *IL1B *mRNA was detected in the spleen, cortex, cerebellum, and brain stem of most pediatric patients that died of cerebral malaria, but it was not detected in those of uninfected individuals [3]. In addition, the concentration of IL-1 receptor antagonist (IL-1RA), which competes for receptor binding with IL-1, was also found to be increased in Gambian children with cerebral malaria [4]. These observations raise the question of whether polymorphisms in the genes encoding IL-1β (*IL1B*) and IL-1RA (*IL1RA*) are associated with cerebral malaria.

*IL1 *family genes including *IL1B *and *IL1RA *form a cytokine gene cluster on human chromosome 2. Recently, the *IL1B *3953C>T polymorphism, which lies in exon 5 of *IL1B*, was reported to be associated with cerebral malaria in Gambia [5]. Gyan et al. [6] reported that, in Ghanaian Children, levels of parasitemia were significantly higher in uncomplicated malaria patients possessing the *IL1B *3953T allele (*i.e*., 3953T/T and 3953C/T genotypes) than those possessing only 3953C (*i.e*., 3953C/C genotype), although this polymorphism and the *IL1RA *variable number of tandem repeat (VNTR) polymorphism were not associated with cerebral malaria.

The functional significance of the *IL1B *3953C>T polymorphism has been studied. Some results show an association of *IL1B *3953C>T with the levels of IL-1 production [7,8], whereas others show no association [9,10]. Recently, Kimura et al. [11] employed allele-specific transcript quantification to examine the effects of *IL1B *-31C>T and 3953C>T polymorphisms on the level of IL-1B transcription. Their results revealed that expression of the -31T allele was significantly higher than that of the -31C allele, while *IL1B *3953C>T did not affect the expression of *IL1B*. The increased level of -31T expression corresponds with the observation that the -31T allele constitutes a TATA sequence in the *IL1B *promoter and induces formation of the transcription initiation complex [12]. Therefore, *IL1B *-31C>T is an important candidate polymorphism that may influence the susceptibility or resistance to cerebral malaria through the upregulation of IL-1β. In addition, the *IL1RA *VNTR polymorphism influences the plasma IL-1RA levels [13], and IL-1RA modulates IL-1 production [14], although there was no significant association of *IL1B *-31C>T and *IL1RA *VNTR with cerebral malaria in Gambia [5]. In this study, *IL1B *-31C>T, *IL1B *3953C>T, and *IL1RA *VNTR polymorphisms were analysed in Thai malaria patients to examine the possible association of these polymorphisms with cerebral malaria.

## Materials and methods

### Subjects

A total of 316 adult patients with *P. falciparum *malaria (110 cerebral malaria and 206 mild malaria patients) living in northwest Thailand were recruited in this study. All of them underwent treatment at the Hospital for Tropical Diseases, Faculty of Tropical Medicine, Mahidol University. Clinical manifestations of malaria have been described previously [15]. All individuals were 13 years of age or older, and the mean ages of the mild malaria and cerebral malaria patients were 25.5 and 28.6, respectively. This study was approved by the institute review board of the Faculty of Tropical Medicine, Mahidol University and the Faculty of Medicine, The University of Tokyo. Informed written consent was obtained from all patients.

### Genotyping

Genomic DNA was extracted from peripheral blood leukocytes using a QIAamp Blood Kit (Qiagen, Hilden, Germany). Genotyping for the three polymorphisms was performed as previously described [11,16]. For the *IL1B *promoter polymorphism, *IL1B *-31C>T [11], PCR was carried out with primers IL1B5'F (5'-TAGTCCCCTCCCCTAAGAACG-3') and IL1Bint1R (5'-CCCAGAATATTTCCCGAGTCA-3') to amplify the region including *IL1B *-31C>T, and the PCR products were treated with *Alu*I (New England Biolabs, Beverly, CA, USA), which digests the -31T allele. For the *IL1B *exon 5 polymorphism, *IL1B 3953C>T *[11], PCR was carried out with primers IL1Bint4F (5'-GCTCAGGTGTCCTCCAAGAAA-3') and IL1Bint5R (5'-GGCCAGTGCAATCAAATGTG-3'), and the PCR products were treated with *Taq*I (New England Biolabs), which digests the 3953C allele. For the *IL-1RA *VNTR polymorphism [16], the region including variable numbers of identical 86-bp tandem repeats was amplified by PCR using the following primers: 5'-CTCAGCCAACACTCCTAT-3' and 5'-TCCTGGTCTGCAGGTAA-3'. PCR products of 240(allele 2, two repeats), 325 (allele 3, three repeats), 410 (allele 4, four repeats), and 500 bp (allele 5, five repeats) were distinguished by agarose gel electrophoresis.

### Statistical analysis

Deviation from Hardy-Weinberg equilibrium was examined with Arlequin software [17] with the default setting, where the exact P-value is calculated based on the Markov chain method [18]. Linkage disequilibrium (LD) between *IL1B *-31C>T and *IL1RA *VNTR was evaluated based on the expectation maximization (EM) algorithm using the Arlequin software with the default setting, where a permutation procedure is performed to calculate the P-value for the likelihood ratio test [19]. Fisher's exact test was performed to compare the genotype and allele frequency distributions between cerebral and mild malaria patients. The P-values for association of *IL1B *-31C>T with cerebral malaria were calculated based on 3 × 2 and 2 × 2 contingency tables for genotype and allele frequency distributions, respectively. The P-values for association of *IL1RA *VNTR with cerebral malaria were calculated based on 5 × 2 and 4 × 2 contingency tables for genotype and allele frequency distributions, respectively. Furthermore, Fisher's exact test was carried out for the comparison of each genotype frequency between mild and cerebral malaria patients based on 2 × 2 table for *IL1B *-31C>T and *IL1RA *VNTR. The frequencies of haplotype consisting of *IL1B *-31C>T and *IL1RA *VNTR were estimated based on EM algorithm [19] using Arlequin software [17]. The difference in haplotype frequency distribution between mild and cerebral malaria patiens was assessed by a likelihood ratio test. In the EM algorithm for the estimation of haplotype frequency, the logarithm of likelihood of the sample, ln *L*, was maximized in each subject group (i.e., ln*L*_m _for mild malaria subjects, ln*L*_c _for cerebral malaria subjects, and ln*L*_all _for the combined subjects). The likelihood-ratio statistic was defined by *LR *= 2 [ln*L*_m _+ ln*L*_c _- ln*L*_all_], which had an approximate χ^2 ^distribution with 7 degrees of freedom under the null hypothesis of no difference in haplotype frequency distribution. It should be noted here that there were 8 possible haplotypes, thus the degree of freedom was 7 in the analysis. The P-values for association of genotypes of *IL1B *-31C>T and *TNFA *-308G>A with cerebral malaria were calculated by Fisher's exact test based on 2 × 2 contingency table for each genotype.

## Results

Because only the 3953C/C genotype was found in 16 patients with mild malaria and 16 patients with cerebral malaria in the polymorphism screening, a further genotyping was not performed. The genotype and allele frequencies of the *IL1B *-31C>T and *IL1RA *VNTR polymorphisms in Thai malaria patients are shown in Tables 1 and 2. It should be noted that some patients were not genotyped for both *IL1B *-31C>T and *IL1RA *VNTR because the target fragment was not amplified by PCR. In Thai malaria patients, the population frequencies of the -31C and -31T alleles were nearly equal, and allele 4 (with four repeats) was predominant in the *IL1RN *VNTR polymorphism. Genotype frequencies did not deviate from predictions of the Hardy-Weinberg equilibrium either in *IL1B *-31C>T (for mild malaria patients, P = 0.58; for cerebral malaria patients, P = 0.44) or in *IL1RA *VNTR (for mild malaria patients, P = 0.27; for cerebral malaria patients, P = 0.73). Significant differences in genotype and allele frequency distributions were not observed between mild and cerebral malaria patients for *IL1B *-31C>T or *IL1RA *VNTR (Tables 1 and 2). In addition, there was no difference in each genotype frequency between mild and cerebral malaria patients (data not shown).

**Table 1:** Genotype and allele frequencies of *IL1B *-31C/T polymorphism in Thai malaria patients.

	Mild malaria (n = 192)	Cerebral malaria (n = 106)	Association P-value^a^
			
Genotype			
*IL1B *-31C>T			
C/C	55 (0.286)	28 (0.264)	0.82
C/T	91 (0.474)	49 (0.462)	
T/T	46 (0.240)	29 (0.274)	
Allele			
*IL1B *-31C>T			
C	201 (0.523)	105 (0.495)	0.55
T	183 (0.477)	107 (0.505)	

Frequencies are in parentheses.

^a^P-values were calculated by the Fisher's exact test based on 3 × 2 and 2 × 2 contingency tables for genotype and allele frequency, respectively.

**Table 2:** Genotype and allele frequencies of *IL1RA *VNTR polymorphism in Thai malaria patients.

	Mild malaria (n = 203)	Cerebral malaria (n = 109)	Association P-value^a^
			
Genotype			
2/2^b^	2 (0.010)	2 (0.018)	0.88
2/4	42 (0.207)	24 (0.220)	
3/4	3 (0.015)	1 (0.009)	
4/4	154 (0.759)	82 (0.752)	
4/5	2 (0.010)	0 (0.000)	
			
Allele			
*IL1RA*			
2	46 (0.113)	28 (0.128)	0.84
3	3 (0.007)	1 (0.005)	
4	355 (0.874)	189 (0.867)	
5	2 (0.005)	0 (0.000)	

Frequencies are in parentheses.

^a ^P-values were calculated by the Fisher's exact test based on 5 × 2 and 4 × 2 contingency tables for genotype and allele frequency, respectively.

^b^It should be noted here that allele with n repeats of VNTR is designated as "n" in this table.

The LD between *IL1B *-31C>T and *IL1RA *VNTR was not strong in the studied population (for mild malaria patients, P = 0.14 by likelihood ratio test based on a permutation precedure; for cerebral malaria patients, P = 0.50 by likelihood ratio test based on a permutation precedure). The estimated frequencies of haplotype consisting of *IL1B *-31C>T and *IL1RA *VNTR polymorphisms were shown in Table 3. No significant difference in the estimated haplotype frequency distribution was detected by a likelihood ratio test (P = 0.60).

**Table 3:** Estimated haplotype frequencies of *IL1B *-31C>T and *IL1RA *VNTR polymorphisms in Thai malaria patients.

Haplotype^a^	Mild malaria	Cerebral malaria	Association P-value^b^
*IL1B *– *IL1RA*			
-31C - 2^c^	0.042	0.046	0.60
-31T - 2	0.072	0.084	
-31C - 4	0.432	0.454	
-31T - 4	0.443	0.414	

^a^Only haplotypes with the estimated haplotype frequency of more than 0.01 were presented.

^b^P-value was calculated by a likelihood ratio test.

^c^It should be noted here that allele with n repeats of VNTR is designated as "n" in this table.

Because several association tests were performed for *IL1B *-31C>T, *IL1RA *VNTR, or the haplotype, the obtained P-values should be corrected based on the number of testings by using a proper method (e.g., Bonferroni's correction). However, none of raw P-values for the association tests were less than 0.05. Thus, the corrected P-values are not presented in this study.

## Discussion

In this study, possible association of *IL1B *-31C>T and *IL1RA *VNTR polymorphisms with cerebral malaria was assessed in 316 Thai malaria patients. These polymorphisms were found not to be associated with cerebral malaria. If the increased expression of IL-1B observed in brain of cerebral malaria patients [2,3] is due to the increased transcription of *IL1B *caused by the *IL1B *-31T allele [11], the frequency of *IL1B *-31T should be higher in cerebral malaria patients than in mild malaria patients. However, the allele frequency of *IL1B *-31T was not significantly higher in the cerebral malaria patients. Because the allele frequency of *IL1B *-31T is approximately 0.5 in the studied population, the present study yields a statistical power high enough for the detection of an association between *IL1B *-31T and cerebral malaria unless the association is very weak. For example, according to the formurae for calculation of statistical power developed by Ohashi et al. [20], when the penetrances for the onset of cerebral malaria are 0.04 for -31T/-31T, 0.02 for -31C/-31T, and 0.01 for -31C/-31C, the estimated power of this study with the significance level of 0.05 exceeds 0.98.

The plasma level of IL1-RA was influenced by the number of repeats in the *IL1RA *VNTR [13], and the allele with two repeats (designated as allele 2 in the present study) has been reported to be associated with various diseases. Like *IL1B *-31T, our study yields a high statistical power for allele 2 of *IL1RA *VNTR if the association is not weak. However, neither *IL1B *-31C>T nor *IL1RA *VNTR showed any association with cerebral malaria. Furthermore, genotype combinations of these polymorphisms were not associated with cerebral malaria (data not shown). The present results are consistent with previous ones in Gambia, where these polymorphisms are not associated with cerebral malaria [5]. Taken together, we conclude that the *IL1B *-31C>T and *IL1RA *VNTR polymorphisms do not play a crucial role in susceptibility or resistance to cerebral malaria.

IL-1 is known to synergize with tumor necrosis factor-α (TNF-α), and their serum levels are thought to play a role in the severity of malaria [21]. The promoter allele of TNF-α gene (*TNFA*), *TNFA *-308A, was reported to be associated with susceptibility to cerebral malaria in Gambia [22]. Although *TNFA *-308A was not associated with cerebral malaria in our previous study for the same Thai malaria patients [23], it is interesting to examine the possible association of combined genotypes of *IL1B *-31C>T and *TNFA *-308G>A polymorphisms with cerebral malaria. Table 4 shows the frequencies of nine combined genotypes of *IL1B *-31C>T and *TNFA *-308G>A in mild and cerebral malaria patients. There was no significant difference in each genotype frequency between mild and cerebral malaria patients, suggests that there is no interactive effect of two polymorphisms on the risk of cerebral malaria.

**Table 4:** Genotypes of *IL1B *-31C>T and *TNFA *-308G>A in Thai malaria patients.

	TNFA -308G>A		
	
	G/G	G/A	A/A
			
Mild malaria (n = 192)			
*IL1B *-31C>T			
C/C^b^	48 (0.250)	7 (0.036)	0 (0.000)
C/T	85 (0.443)	6 (0.031)	0 (0.000)
T/T	43 (0.224)	3 (0.016)	0 (0.000)
			
Cerebralmalaria (n = 105)			
*IL1B *-31C>T			
C/C	25 (0.238)	2 (0.019)	0 (0.000)
C/T	41 (0.390)	8 (0.076)	0 (0.000)
T/T	25 (0.238)	3 (0.029)	1 (0.009)

Frequencies are in parentheses.

There was no significant difference in each genotype frequency between mild and cerebral malaria by the Fisher's exact test based on 2 × 2 contingency table where all the other genotypes are combined.

*IL1B *and *IL1RA *are located closely on human chromosome 2. Although *IL1RA *VNTR is in strong LD with *IL1B *polymorphisms, such as *IL1B *-511C>T, *IL1B *-31C>T, and *IL1B *3953C>T in Caucasian populations [12,10,21], significant LD was not observed between *IL1RA *VNTR and *IL1B *-31C>T in the Thai population. In Indonesians, LD was not found even between *IL1B *-31C>T and 3953C>T [11]. These observations suggest that the structure of LD in the *IL1 *gene cluster differs among populations. Because significant LD was not observed between *IL1B *and *IL1RA *in the present study, we cannot exclude the possibility that polymorphisms in the other *IL1 *family genes located between *IL1B *and *IL1RA*, such as *IL1 *family member 9 (*IL1F9*) and *IL1 *family member 8 isoform 1 (*IL1F8*), are associated with cerebral malaria. It is currently unknown whether associations between *IL1*B 3953C>T and cerebral malaria [5] or parasitemia [6] in African populations result from a LD with other functional polymorphisms in this chromosomal region. Thus, further studies of various populations are required to clarify whether polymorphisms of *IL1 *family genes are involved in the pathogenesis of cerebral malaria.

## Authors' contributions

S. Looareesuwan and N. Tangpukdee diagnosed malaria and collected blood samples. J. Patarapotikul and H. Hananantachai extracted DNA from the blood samples. J. Ohashi and J. Patarapotikul designed this study. I. Naka performed genotyping with A. Doi. J. Ohashi did statistical analyses and wrote the report. K. Tokunaga and S. Looareesuwan, as scientific coordinators of this project, received the financial support.
